# Human Telomerase RNA Protein Encoded by Telomerase RNA is Involved in Metabolic Responses

**DOI:** 10.3389/fcell.2021.754611

**Published:** 2021-12-07

**Authors:** Viktoriia Shliapina, Mariia Koriagina, Daria Vasilkova, Vadim Govorun, Olga Dontsova, Maria Rubtsova

**Affiliations:** ^1^ Shemyakin-Ovchinnikov Institute of Bioorganic Chemistry of the Russian Academy of Sciences, Moscow, Russia; ^2^ Department of Chemistry and A. N. Belozersky Institute of Physico-Chemical Biology, Lomonosov Moscow State University, Moscow, Russia; ^3^ Federal Research and Clinical Centre of Physical-Chemical Medicine, Moscow, Russia; ^4^ Center of Life Sciences, Skolkovo Institute of Science and Technology, Moscow, Russia

**Keywords:** telomerase, hTERP, metabolism, proliferation, signaling pathway

## Abstract

Cell proliferation is associated with increased energy and nutrients consumption. Metabolism switch from oxidative phosphorylation to glycolysis and telomerase activity are induced during stimulation of proliferation, such as tumorigenesis, immune cell activation, and stem cell differentiation, among others. Telomerase RNA is one of the core components of the telomerase complex and participates in survival mechanisms that are activated under stress conditions. Human telomerase RNA protein (hTERP) is encoded by telomerase RNA and has been recently shown to be involved in autophagy regulation. In this study, we demonstrated the role of hTERP in the modulation of signaling pathways regulating autophagy, protein biosynthesis, and cell proliferation. The AMPK signaling pathway was affected in cells deficient of hTERP and when hTERP was overexpressed. The appearance of hTERP is important for metabolism switching associated with the accelerated proliferation of cells in healthy and pathological processes. These findings demonstrate the connection between telomerase RNA biogenesis and function and signaling pathways.

## Introduction

Cell proliferation requires accelerated production of proteins, lipids, and nucleic acids with the biosynthesis of these components being regulated by a network of metabolic pathways. Most somatic cells do not proliferate, but accelerated proliferation occurs during tumorigenesis, T-cell activation, and stem-cell differentiation. The increased proliferation requires increased consumption of nutrients, which is accompanied by switching from oxidative phosphorylation to glycolysis, a process known as the Warburg effect (Hanahan and Weinberg, 2011; Burgess et al., 2014; Almeida et al., 2016). The stimulation of proliferation and increased rates of cellular division also results in a shortening of telomeres, special structures located at the ends of linear eukaryotic chromosomes. Telomeres contain many copies of TTAGGG repeats associated with protein complex shelterin and are involved in the stabilization and protection of chromosome ends, as well as in the regulation of telomerase binding and telomeric DNA synthesis (Monaghan and Ozanne, 2018). Cell division leads to a progressive loss of telomeres due to incomplete DNA replication (Olovnikov, 1973; Chow et al., 2012) and nuclease activity (Pfeiffer and Lingner, 2012). When telomeres become critically short arrest of senescence and cell death are induced (Fumagalli et al., 2012; Hewitt et al., 2012). Telomerase is a large ribonucleoprotein complex responsible for the synthesis of telomeric DNA repeats, thereby compensating for telomere loss (Lingner et al., 1995). Telomerase is inactivated in the majority of somatic cells and its reactivation is associated with accelerated proliferation during tumorigenesis, T-cell activation, and stem-cell differentiation.

Human telomerase consists of two core components: human telomerase RNA (hTR), also known as human telomerase RNA component (hTERC), and human telomerase reverse transcriptase (hTERT). The expression of *hTERT* correlates with telomerase activity and is regulated at the transcriptional level (Feng et al., 1995; Roake and Artandi, 2020). However, hTERC is present in most somatic cells and it’s amount depends not only on the transcriptional activity of *hTERC*, but also on the turnover of hTERC primary transcripts (Roake and Artandi, 2020). The primary transcript of hTERC is synthesized by RNA polymerase II from a dedicated locus and its own promoter (Blasco et al., 1995; Feng et al., 1995). The hTERC transcript is synthesized as an extended precursor molecule of approximately 461 nucleotides with heterogenous 3′-end, which is processed into the mature 451-nucleotide RNA (Roake et al., 2019). Longer hTERC precursors exceeding 1500 nucleotides in length may represent either a very early hTERC precursor that is rapidly processed into the shorter forms or is targeted for destruction by RNA surveillance (Tseng et al., 2015). The Integrator complex facilitates transcription from the *hTERC* promoter and regulates *hTERC* transcription termination via an unknown mechanism. Depletion of the major Integrator subunit results in the accumulation of an extended transcript of 571 nucleotides (Rubtsova et al., 2019) that functions as a template for synthesis of endogenous human telomerase RNA protein (hTERP). Previously, we detected hTERP in HEK293T cells and demonstrated its protective function under stress conditions (Rubtsova et al., 2018). Although the function of this protein is largely unknown, we have shown that mutations at the N-terminus of hTERP affect basal levels of autophagy. Currently, there is limited data on the function of telomerase components outside the telomerase complex (Rubtsova and Dontsova, 2020). We propose that appearance of different products of biogenesis of hTERC primary transcript may define the metabolic program and level of proliferation of cell.

**FIGURE 1:**
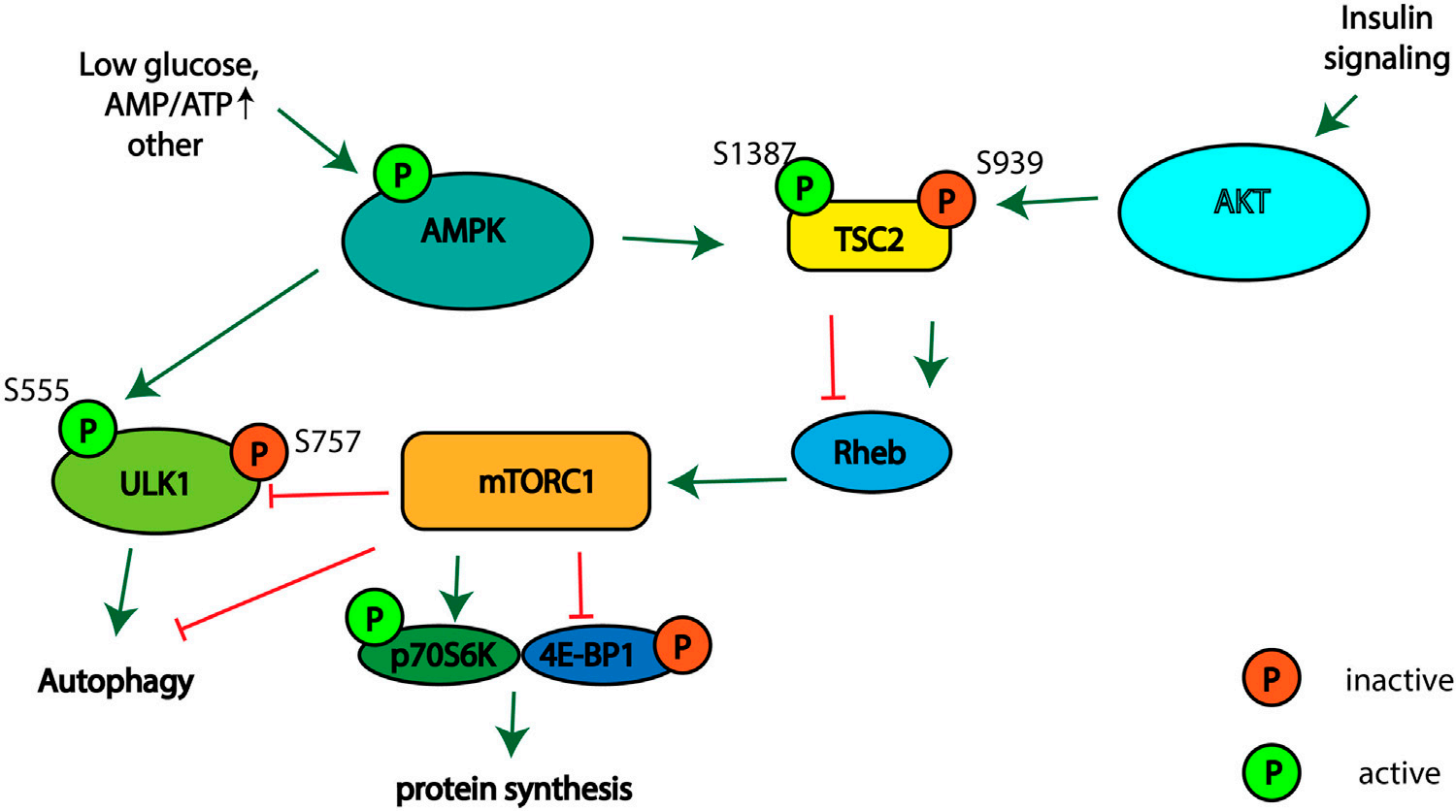
Schematic illustrating the signaling pathways involved in regulation of mTORC1 activity, autophagy, and protein synthesis.

Different signaling pathways regulate autophagy, cellular proliferation, and cell growth. The tuberous sclerosis complex (TSC), which consists of proteins TSC2, TSC1, and TBC197 (Menon et al., 2014), is regulated by AMP-activated protein kinase (AMPK) and RAC-alpha serine/threonine protein kinase (AKT1) signaling in response to different stimuli (Kwiatkowski and Manning, 2005; Huang et al., 2008; Hoxhaj and Manning, 2020). TSC2 acts as a GTPase-activating protein of the small GTPase protein Ras homolog enriched in the brain (Rheb) (Inoki, 2003). GTP-bound Rheb activates mammalian target of rapamycin complex 1 (mTORC1), which plays a conserved role in the regulation of cell growth, proliferation, survival and autophagy activation (Condon and Sabatini, 2019). mTORC1 stimulates cellular growth and proliferation by phosphorylation of ribosomal S6 kinase 1 (p70S6K1) and the translation initiation factor 4E binding protein 1 (4E-BP1), thereby stimulating cap-dependent translation (Holz et al., 2005). Activated mTORC1 phosphorylates and thereby inhibits autophagy activating Unc-51 like autophagy activating kinase 1 (ULK1) (Figure 1). AMPK is involved in autophagy stimulation upon glucose starvation, amino acids deficiency and when ATP levels are low (Hardie et al., 2012; González et al., 2020). AMPK activates TSC2 *via* phosphorylation at Thr1271 and Ser1387 (Inoki et al., 2003). TSC2 activation leads to the suppression of Rheb activity, and as a result to mTORC1 inhibition. Thus, the activation of AMPK leads to the inhibition of protein synthesis and cell proliferation and stimulation of autophagy. The activity of the TSC complex is also regulated by the phosphorylation of TSC2 at various sites by AKT-kinase involved in the cellular response to growth factors, DNA damage, the regulation of glucose metabolism and glycolysis in cancer cells (Skeen et al., 2006; Hoxhaj and Manning, 2020). Activation of AKT leads to phosphorylation of TSC2 at Ser939, which stimulates mTORC1 activity and inhibits autophagy (Dibble and Cantley, 2015). Thus, the activation of AMPK leads to the inhibition of protein synthesis and cell proliferation, and the stimulation of autophagy. Meanwhile, activation of the AKT signaling pathway has an opposite effect on stimulating protein synthesis and cell proliferation and inhibiting autophagy.

In the current study, we analyzed the level of basal autophagy and the status of various signaling pathways in hTERP-deficient and hTERP-overexpressing cells under various conditions affecting cellular metabolism in order to demonstrate the connection between telomerase RNA biogenesis and function and signaling pathways.

## Materials and Methods

### Cell Culture

Human HEK293T and U2OS cells were grown in DMEM/F12 medium supplemented with Glutamax (Thermo Fisher Scientific), 10% fetal bovine serum (FBS), 100 units/mL penicillin, and 100 μg/ml streptomycin at 37°C and 5% CO2. Cultures were examined under an inverted microscope to determine confluency and viability. The cells were confirmed to be negative for *mycoplasma* contamination.

To generate an hTERP open reading frame (ORF) knockout HEK293T cell line (dhTERP), we applied CRISPR/Cas9 technology as previously (Rubtsova et al., 2018). Briefly, oligonucleotides corresponding to the single guide RNA (sgRNA) sequence after hybridization were ligated with the pX458 vector (Ran et al., 2013) digested with BbsI endonuclease. The obtained plasmid was confirmed by sequencing and transfected into HEK293T cells using Lipofectamine 3000 reagent. Cells transfected with plasmids and expressing green fluorescent protein (GFP) allowed us to enrich and clone the population by cell sorting using a FACSAriaIII flow cytometer (BD). Analysis of the monoclones revealed only 1 cell line with a full knockout of the hTERP ORF, which was confirmed by PCR-amplification followed by sequencing of obtained product. To generate cell lines overexpressing hTERP fused with a C-terminal 3HA tag (hTERP-3HA) or 3HA alone as a control, we cloned the corresponding coding sequences into pSBtet-Neo vector (Kowarz et al., 2015) digested initially with SfiI endonuclease. The obtained plasmid was confirmed by sequencing and transfected into cells using Lipofectamine 3000 reagent. Cells after transfection were cultivated in a medium containing G418 antibiotic for selection of cells containing the expression cassettes. Cell lines with a doxycycline-induced expression of hTERP-3HA or only 3HA were cultivated in a medium containing G418 antibiotic for selection of cells expressing the cassettes. Selected cells were treated with doxycycline to induce the expression of hTERP-3HA and 3HA.

### Cell Treatment

Cells were seeded at 3.0 × 10^3^/cm^2^ into a 6-well plate and treated with 10 µM chloroquine (cat. #C66288, Sigma-Aldrich) for 6 h to inhibit autophagy progression or with the adenosine analog 2 mM 5-aminoimidazole-4-carboxamide ribonucleotide (AICAR; 9944, Cell Signaling Technology) for 1 h to stimulate AMPK activity. For amino acid starvation, cells were incubated with medium lacking amino acids (20 mM HEPES, pH 7.4; 140 mM NaCl; 1 mM CaCl_2_; 1 mM MgCl_2_; 5mM glucose) for 1 h at 37°C with or without chloroquine. Inhibition of glycolysis was performed by treating the cells with 2 mM 2-deoxy-D-glucose (2-DG) for 48 h.

### Immunoblotting

Cells were rinsed with cold phosphate-buffered saline (PBS) and lysed with NETN buffer (150 mM NaCl; 1 mM EDTA; 50 mM Tris-HCl, pH 7.5; 0.5% NP-40) containing Halt Protease and Phosphatase Inhibitor Cocktail (Promega) for 30 min on ice. The lysates were then sonicated for 15 s and centrifuged at 14 000 × *g* for 10 min at 4°C. Protein concentrations were determined by measuring the optical density (OD) at absorbance of 280 nm (A_280_) using a NanoDrop 2000 spectrophotometer (Thermo Fisher). Equal amounts of protein extracts (20 µg) were boiled for 5 min in NuPAGE LDS sample buffer (Life Technologies) and the proteins separated on NuPAGE 4–12% Bis-Tris gels (Life Technologies) under denaturing conditions when we analyzed the phosphorylation status. The proteins were separated in 15% PAGE under denaturing conditions for analysis of LC3I protein conversion to LC3II form. Immunoblotting was performed according to standard methods and analyzed using the following primary antibodies: anti-MAP LC3 alpha/beta (cat. #sc-398822; Santa Cruz Biotech), anti-AKT (cat. #9272; Cell Signaling Technology), anti-phospho-AKT (Thr308) (cat. #4056; Cell Signaling Technology), anti-AMPK alpha (cat. #5832; Cell Signaling Technology), anti-phospho-AMPK alpha (Thr172) (cat. #2535; Cell Signaling Technology), anti-p70S6K (cat. #9202; Cell Signaling Technology), anti-phospho-p70S6K (Thr389) (cat. #9206; Cell Signaling Technology), anti-TSC2 (cat. #4308; Cell Signaling Technology), anti-phospho-TSC2 (Ser1387) (cat. #5584; Cell Signaling Technology), anti-phospho-TSC2 (Ser939) (cat. #3615; Cell Signaling Technology), anti-ULK1 (cat. #8054; Cell Signaling Technology), anti-phospho-ULK1(Ser555) (cat. #5869; Cell Signaling Technology), anti-phospho-ULK1(Ser757) (cat. #6888; Cell Signaling Technology), anti-4E-BP1 (cat. #9452; Cell Signaling Technology), anti-phospho-4E-BP1(Thr37/46) (cat. #2855; Cell Signaling Technology), anti-HA-HRP (3F10) (cat. #12 013 819 001; Roche), anti-GAPDH (cat. #Ab9485; Abcam). The HRP-conjugated secondary antibodies included anti-mouse (cat. #G2-6520; Thermo Fisher), anti-goat (cat. #G1-1620; Thermo Fisher), and anti-rabbit (cat. #7074; Cell Signaling Technology). We used previously obtained polyclonal anti-hTERP antibodies (Rubtsova et al., 2018) for analysis of hTERP level.

### Statistical Analysis

Statistical analysis was performed using GraphPad Prism 7.0 software (GraphPad, La Jolla, CA, United States). Statistical significance was determined using one-way and two-way analysis of variance (ANOVA) and differences between the analyzed samples were determined using Sidak’s or Dunett’s multiple comparison tests. Each experiment was repeated at least three times.

## Results

### hTERP is Involved in Regulation of Induced Autophagy

We previously demonstrated that mutations in the N-terminus of hTERP affect basal levels of autophagy (Rubtsova et al., 2018). Here, we aimed to determine the precise step of the autophagic process at which the inhibition occurs. To investigate whether hTERP is involved in the regulation of signaling pathways and any potential mechanisms, we used the hTERP-deficient HEK293T cell line generated by CRISPR/Cas9 genome editing and the telomerase-positive HEK293T or telomerase-negative U2OS cell lines overexpressing hTERP that were obtained using the Sleeping Beauty transposase system (Kowarz et al., 2015). CRISPR-Cas9 treatment results in the absence of 5 nucleotides after start AUG-codon (Supplementary Figure S1A) that entailed the ORF disturbance and the absence of hTERP protein (Supplementary Figure S1B) while the exogenous expression of hTERP ORF increased slightly the level of hTERP protein in the case of wild type HEK293T and restored the basal level of hTERP in knockouted HEK293T cells (Supplementary Figure S1B). The expression of hTERP-3HA was confirmed by western blotting using antibodies specific to HA-epitope (Supplementary Figures S1C,D) or using antibodies specific to hTERP (Supplementary Figure S1B).

**FIGURE 2:**
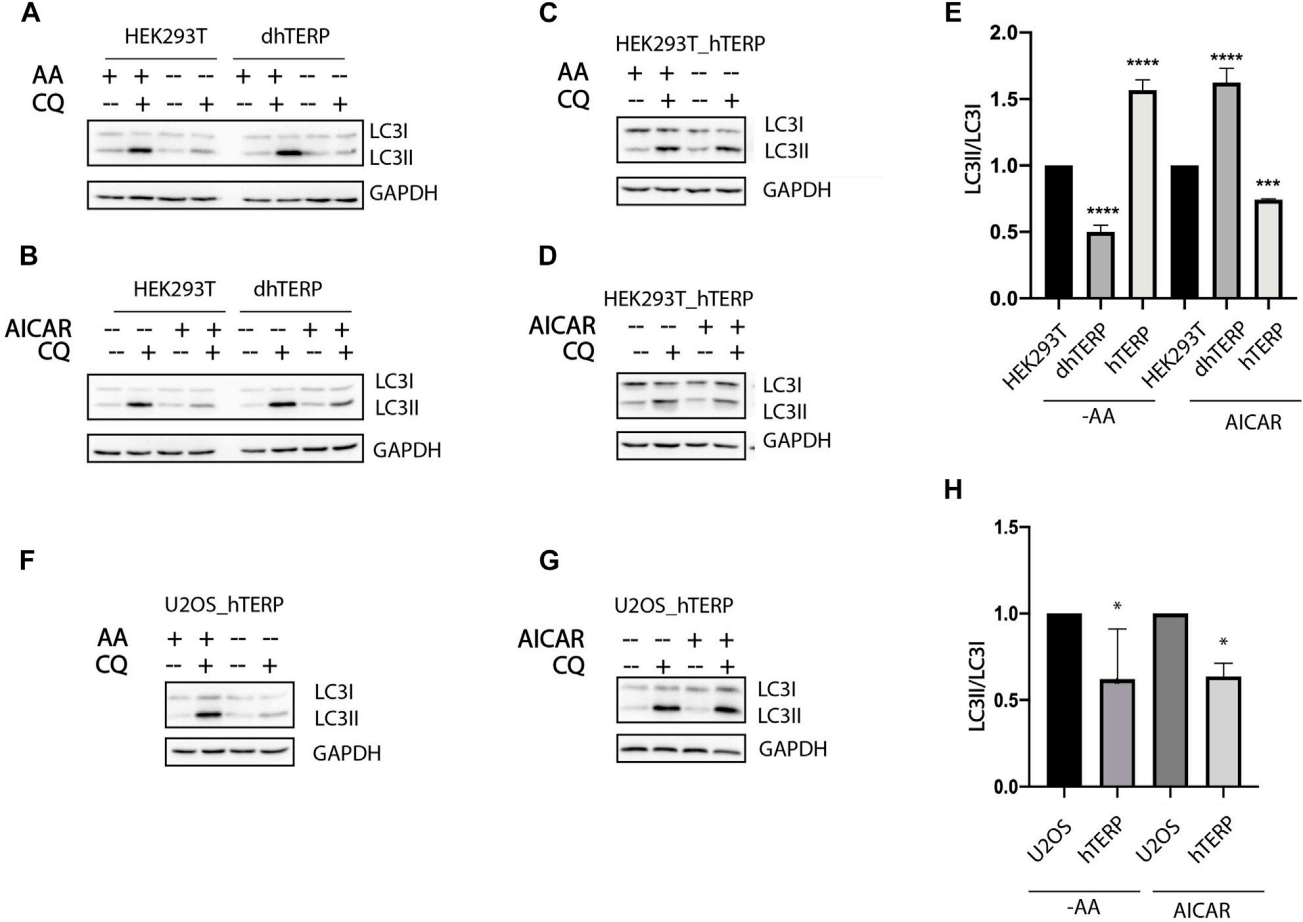
hTERP-deficiency influences autophagy progression. (**A,B**) Immunoblotting of lysates from wild-type and dhTERP HEK293T cells for LC3 under normal conditions (AA+) and during amino acids starvation (AA−) **(A)** and under normal conditions and AICAR treatment **(B)**. To block autophagy progression, cells were treated with 10 µM chloroquine (CQ). GAPDH was used as loading control. (**C,D**) Immunoblotting of lysates from wild-type and overexpressing-hTERP-3HA HEK293T cells for LC3 under normal conditions (AA+) and during amino acids starvation (AA−) **(C)** and under normal conditions and AICAR treatment **(D)**. **(E)** Quantification of the conversion rate of LC3-I to LC3-II in wild type, dhTERP and overexpressing hTERP HEK293T cells starved of amino acids or treated with AICAR. The ratio of LC3-II to LC3-I in chloroquine treated amino acids starved cells **(A,C)** or cells under AICAR treatment **(B,D)** was normalized to the ratio of LC3-II to LC3-I in chloroquine treated cells cultivated in normal conditions. Conversion rate of LC3 for mutant cells was normalized to the same ratio for wild type cells. The conversion rate of LC3-I to LC3-II was determined from three independent experiments (mean ± SEM) and quantified using GraphPad Software. ***p < 0,001 by Dunett’s multiple comparison test. **(F,G)** Immunoblotting of lysates from wild-type and overexpressing hTERP-3HA U2OS cells for LC3 under normal conditions (AA+) and during amino-acids starvation (AA−) **(F)** and under normal conditions or AICAR treatment **(G)**. **(H)** Quantification of the conversion rate of LC3-I to LC3-II in wild type and overexpressing hTERP U2OS cells starved of amino acids or treated with AICAR. The ratio of LC3-II to LC3-I in chloroquine treated amino acids starved cells **(F)** or cells under AICAR treatment **(G)** was normalized to the ratio of LC3-II to LC3-I in chloroquine treated cells cultivated in normal conditions. Conversion rate of LC3 for mutant cells was normalized to the same ratio for wild type cells. The conversion rate of LC3-I to LC3-II was determined from three independent experiments (mean ± SEM) and quantified using GraphPad Software. **p < 0.01 and *p < 0,05 by Dunett’s multiple comparison test.

To investigate the role of hTERP in autophagy progression we subjected cells to amino acids starvation or to treatment with 5-aminoimidazole-4-carboxamide ribonucleotide (AICAR) (Figures 2). Both treatments regulate signaling pathways related to autophagy initiation and regulation of cell proliferation. The conversion of LC3-I to LC3-II as a result of conjugation of phosphatidylethanolamine to LC3-I during authophagy was analyzed by western blotting when autophagosome-lysosome fusion was blocked by chloroquine (Kabeya, 2000). We found that amino acids starvation resulted in the inhibition of autophagy while AICAR treatment activated the autophagy in hTERP-deficient cells (Figures 2A,B,E). Overexpression of hTERP in HEK293T cells (Supplementary Figures S1B, S1C) activated autophagy in amino acids starved cells (Figures 2C,E) and inhibited it in cells treated with AICAR (Figures 2D,E). Overexpression of hTERP in the U2OS cells (Supplementary Figure S1D) resulted in inhibition of autophagy under amino acids starvation (Figures 2F,H) and after AICAR treatment (Figures 2G,H).

### hTERP Modulates AMPK, TSC2, and ULK1 Phosphorylation Under Glycolysis Inhibition

**FIGURE 3:**
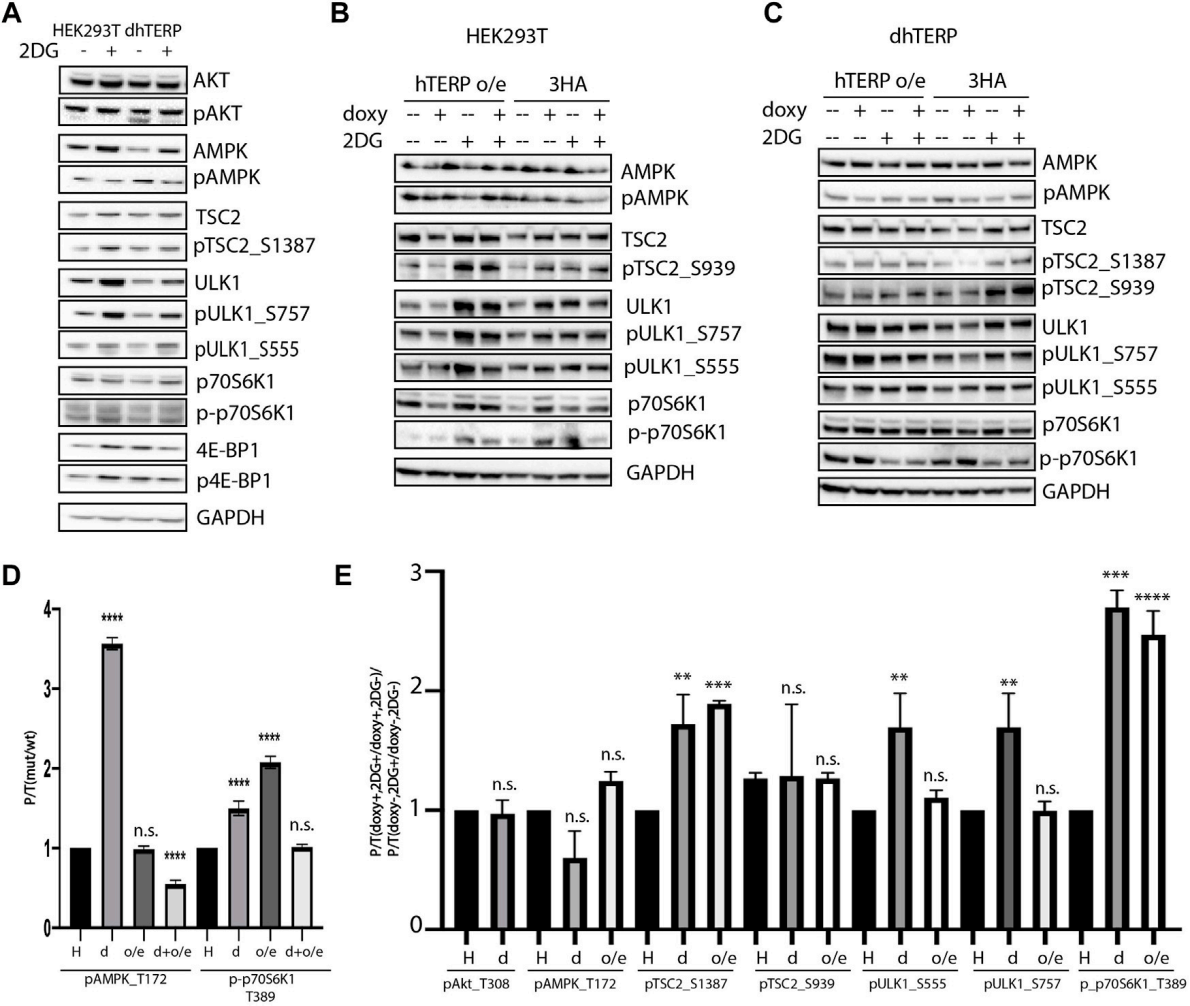
hTERP modulates activity of kinases of relevant signaling pathways in HEK293T cells. **(A)** Lysates from wild-type and dhTERP HEK293T cells with or without 2 mM 2-deoxy-D-glucose (2DG) were probed with the indicated antibodies. **(B,C)**. Lysates prepared from wild-type HEK293T cells **(B)** and dhTERP HEK293T cells **(C)** exogenously expressing 3HA (HA) or hTERP-3HA (hTERP o/e) with or without 2 mM 2-DG were probed with the indicated antibodies. **(D)** Quantification of phosphorylation levels of the indicated proteins. Black bars correspond to the ratio of phosphorylated protein to total protein levels in wild-type HEK293T cells. Grey bars correspond to the ratio of phosphorylated protein to total protein level in dhTERP cells. Dark grey bars correspond to the ratio of phosphorylated protein to total protein level in overexpressing hTERP HEK293T cells treated with doxycycline. Light grey bars correspond to the ratio of phosphorylated protein to total protein level in overexpressing hTERP dhTERP HEK293T cells treated with doxycycline. The ratio for modified cells was normalized to the ratio for wild type HEK293T cells from three independent western blots (mean ± SEM) and quantified using GraphPad software. ****p < 0.0001 by Dunett’s multiple comparison test. **(E)** Quantification of phosphorylation levels of the indicated proteins. Black bars correspond to the ratio of phosphorylated protein to total protein levels in wild-type HEK293T cells treated with 2-DG compared to that of the ratio in untreated cells. Grey bars correspond to the ratio of phosphorylated protein to total protein levels in dhTERP cells treated with 2-DG compared to that of the ratio in untreated cells. White bars correspond to the ratio of phosphorylated protein to total protein levels in overexpressing hTERP dhTERP HEK293T cells treated with doxycycline (doxy+), or 2-DG normalized to the ratio for overexpressing-3HA dhTERP HEK293T cells treated with doxycycline (2DG+/doxy+), or cells not treated with 2-DG (2DG−). The same quantification was performed for samples not treated with doxycycline (doxy−), or treated with 2-DG relative to the ratio of cells not treated with doxycycline (2DG+/doxy−), or cells not treated with 2-DG (2DG−). The normalized data were used for quantification of changes in the phosphorylation rate of targeted protein upon the exogenous expression of hTERP [(doxy+, 2DG+/doxy+, 2DG−)/(doxy−, 2DG+/doxy−, 2DG−)]. The ratio for cells treated with 2-DG was normalized to the ratio for untreated cells from three independent western blots (mean ± SEM) and quantified using GraphPad software. **p < 0.01, ***p < 0.001, and ****p < 0.0001 by Dunett’s multiple comparison test.

To compare the activity of the kinases that participate in signaling pathways regulating mTORC1 activity in cells where hTERP was deleted (Figure 3A) or overexpressed (Figure 3B), we calculated the ratios of phosphorylated kinase to total kinase in cells at normal conditions (Figure 3D). We observed that hTERP level influences the phosphorylation of AMPK and p70S6K1 significantly (Figure 3D). Interestingly, both depletion and overexpression of hTERP result in enhanced phosphorylation of p70S6K1 (Figure 3D). The level of phosphorylated p70S6K1 in knockouted cells restored to wild type HEK293T cells after exogenous expression of hTERP (Figures 3C,D). The level of phosphorylation of AMPK was increased in cells without hTERP and exogenous expression of hTERP in knockouted cells restored the phosphorylation of AMPK to wild type level. Overexpression of hTERP in wild type HEK293T cells didn’t influence on AMPK modification.

Both treatments (amino acids starvation and AICAR treatment) that we used to analyze the influence of hTERP on basal autophagy modulate the AMPK activity (Hardie et al., 2012; González et al., 2020). To explore the possible mechanisms of hTERP action on processes that regulate cellular responses to stressors, we decided to modulate the inhibition of glycolysis by treating the cells with 2-deoxy-D-glucose (2-DG) that also influences AMPK activity.

**FIGURE 4:**
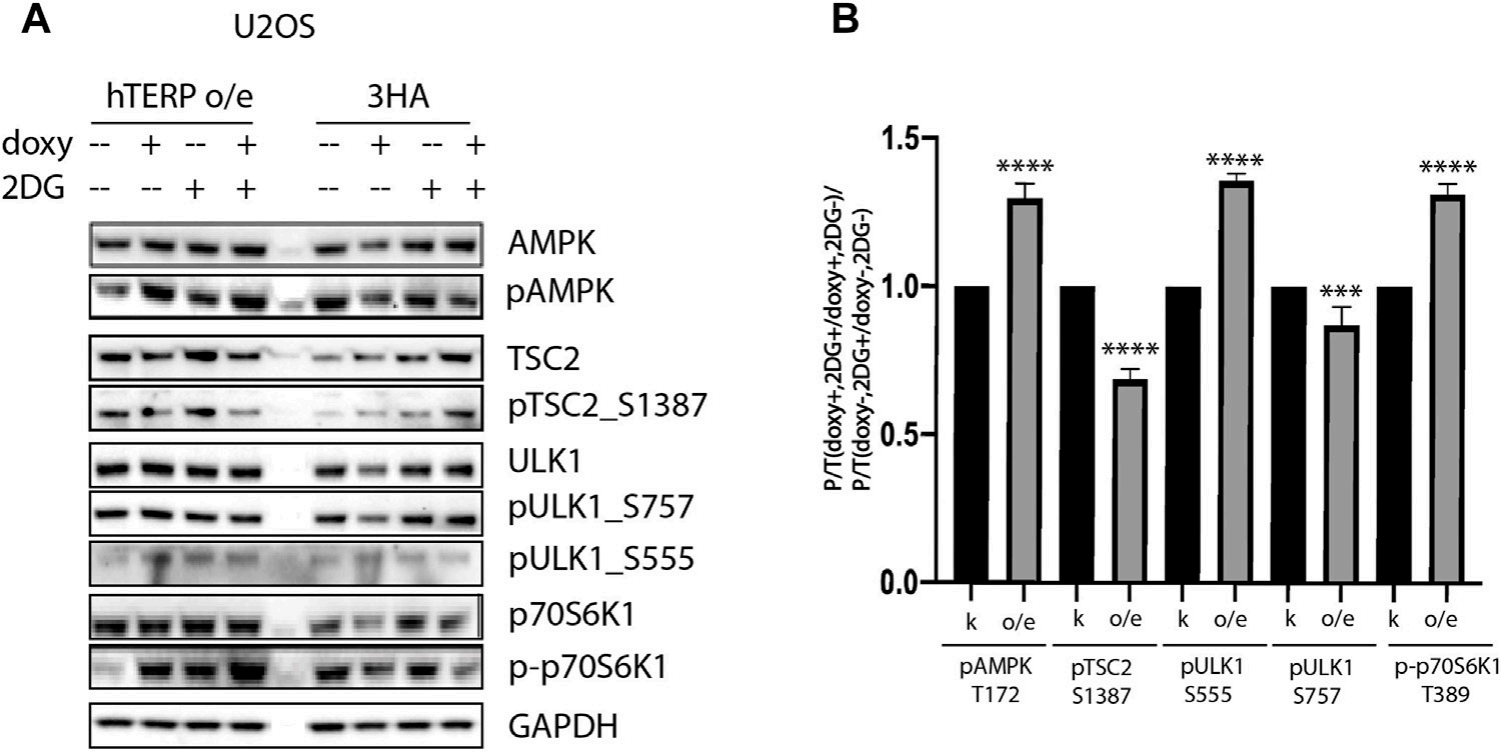
hTERP modulates activity of kinases of relevant signaling pathways in U2OS cells. **(A)** Lysates prepared from U2OS cells exogenously expressing 3HA or hTERP-3HA treated with or without 2 mM 2-DG were probed with the indicated antibodies. **(B)** Quantification of phosphorylation levels of the indicated proteins. Black bars correspond to ratio of phosphorylated protein to total protein levels in overexpressing-3HA U2OS cells treated with doxycycline (doxy+), or 2-DG treated cells normalized to the ratio for cells treated with doxycycline (2DG+/doxy+), cells not treated with 2-DG (2DG−). Grey bars correspond to ratio of phosphorylated protein to total protein levels in overexpressing-hTERP cells treated with doxycycline (doxy+), or 2-DG treated cells normalized to the ratio for cells treated with doxycycline (2DG+/doxy+), or cells untreated with 2-DG (2DG−). The same quantification was performed for the samples not treated with doxycycline (doxy−), or treated with 2-DG relative to the ratio of cells not treated with doxycycline (2DG+/doxy−), or cells not treated with 2-DG (2DG−). Those normalized data were used for quantification of changes in the phosphorylation rate of targeted protein upon the exogenous expression of hTERP [(doxy+, 2DG+/doxy+, 2DG−)/(doxy−, 2DG+/doxy−, 2DG−)]. The ratio for cells treated with 2-DG was normalized to the ratio for untreated cells from three independent western blots (mean ± SEM) and quantified using GraphPad software. ***p < 0.001, and ****p < 0.0001 by Dunett’s multiple comparison test.

To confirm the involvement of hTERP in the regulation of kinase activity, we analyzed their phosphorylation status in wild-type and mutant HEK293T cells (Figures 3A–C) and wild type U2OS cells and U2OS cells overexpressing hTERP (Figure 4A). Cell lines with a doxycycline-induced expression of hTERP-3HA or only 3HA were treated with doxycycline to induce the expression of hTERP-3HA and 3HA and treated with 2 mM 2-DG for 48 h. The expression of hTERP-3HA was confirmed by western blotting using antibodies specific to hTERP and HA (Supplementary Figures S1B–D). Cells treated with only doxycycline or 2-DG were used as controls. Levels of total and phosphorylated forms of the kinases involved in the cellular response to glucose deficiency were analyzed by western blotting (Figures 3A–C, 4A). The ratios of levels of phosphorylated kinase to total kinase obtained for wild-type and dhTERP HEK293T cells treated with 2-DG were normalized to the ratios obtained for untreated cells (Figures 3E, 4B).

We observed that dhTERP HEK293T cells demonstrated reduced activation of AMPK compared with that of wild-type HEK293T cells in response to 2-DG treatment (Figures 3A,E), which was complemented in cells overexpressing hTERP-3HA (Figures 3C,E). Phosphorylation of AMPK was increased in U2OS cells overexpressing hTERP-3HA in response to 2-DG treatment (Figures 4A,B). However, phosphorylation of the direct targets of AMPK, ULK1 Ser555 and TSC2 Ser1387, increased slightly in dhTERP cells compared with that of wild-type HEK293T cells (Figures 3A,E), which should have resulted in the activation of autophagy in hTERP-deficient cells. The levels of phosphorylation of ULK1 at Ser555 and TSC2 at Ser1387 were restored by hTERP-3HA overexpression to levels similar to those observed in the wild-type cells. On the other hand, p70S6K and 4E-BP1 phosphorylation were increased in dhTERP cells (Figure 3A), providing for the activation of protein synthesis and stimulation of cell proliferation. We did not observe any significant changes in the phosphorylation of AKT kinase or its target TSC2 at Ser939 (Figures 3B,C,E). Overexpression of hTERP-3HA in U2OS cells resulted in a slight inhibition of phosphorylation of TSC2 at Ser1387 and ULK1 at Ser757 and increased phosphorylation of pULK1 at Ser555 and p70S6K1 (Figures 4A,B) under 2-DG treatment. As phosphorylation of ULK1 Ser757 inhibits autophagy, hTERP is involved in the regulation of the signaling pathway responsible for sensing cellular energy status. On one hand, hTERP-deficiency led to the activation of protein biosynthesis with increased phosphorylation of p70S6K1 and 4E-BP1, while on the other hand it led to activation of autophagy with increased phosphorylation of ULK1 at Ser555. Notably, we observed the influence of hTERP on the phosphorylation of downstream targets of mTORC1, such as ULK1 Ser757, p70S6K1, and 4E-BP1, suggesting hTERP is involved in regulation of the mTORС1 signaling pathway.

## Discussion

Nutrient starvation results in growth arrest and the activation of autophagy, which allows for the acquisition of deficient resources by digestion of intracellular components. AMPK is a crucial regulator of cellular metabolism in eukaryotes and it regulates cell growth and autophagy (Mihaylova and Shaw, 2011). As a sensor that detects cellular energy status, AMPK is activated when cellular ATP levels are low (Kwiatkowski and Manning, 2005) and its activation has been suggested to prevent cellular senescence and aging (Salminen and Kaarniranta, 2012). Moreover, AMPK activity is regulated by serine-threonine kinase LKB1 tumor suppressor signaling and it is an upstream component of the mTORC1 pathway. AMPK-deficient cells are resistant to oncogenic transformation and tumorigenesis (Shackelford and Shaw, 2009). AMPK and mTORC1 regulate autophagy via ULK1 kinase, an inducer of the autophagy activator complex. The double-negative feedback loop between AMPK and mTORC1, with participation of ULK1, controls the signaling pathway network involved in autophagy regulation (Watanabe et al., 2011). Hyperactivation of mTORC1 leads to the inhibition of AMPK and autophagy (Holczer et al., 2019). Interestingly, ULK1 downregulates AMPK activity via a negative feedback loop that generates a homeostatic response with respect to cellular stress (Löffler et al., 2011). The dephosphorylation of AMPK is crucial in order to prevent the hyperactivation of self-cannibalism, which may have a dramatic effect on the cell (Holczer et al., 2019). The involvement of telomerase components in the regulation of autophagy has also been demonstrated in several studies (Cheng et al., 2015; Harris and Cheng, 2016; Roh et al., 2018; Rubtsova et al., 2018).

The hTERP protein was discovered several years ago and identified as a protein encoded by human telomerase RNA. Full-length protein can be translated from the elongated precursor of telomerase RNA (Rubtsova et al., 2018). Furthermore, it is known that the processing of telomerase RNA is tightly regulated, as the level of telomerase RNA should be constant in order to maintain cellular homeostasis (Tseng et al., 2015). Telomerase RNA is expressed in the majority of somatic cells and is expressed independent of telomerase activity, although at much lower levels in comparison with that of telomerase-positive cells (Artandi et al., 2002; Roake and Artandi, 2020).

The stimulation of cell proliferation is associated with a switch in metabolism from oxidative phosphorylation to glycolysis, which is needed to provide the energy and nutrients necessary for an increased rate of cellular division. The activation of telomerase in cells with an increased proliferative rate results in telomere lengthening, which allows the cells to safely increase the number of divisions. Both hTERT and hTERC are involved in the regulation of regulatory cascades (Ségal-Bendirdjian and Geli, 2019; Rubtsova and Dontsova, 2020). It has been shown previously that hTERC is imported into mitochondria, processed there and released to the cytosol as TERC-53 fragment with unknown function (Cheng et al., 2018). hTERC-53 accumulates in cytoplasm when membrane potential of mitochondria is impaired. It was recently demonstrated that p70S6K1 is persistently phosphorylated and mTORC1 is hyperactivated in *Terc* ^*−/−*^ mice (Ferrara-Romeo et al., 2020). *TERC* knockout leads to the absence of telomerase RNA, which is a component of the telomerase complex that maintains telomeres and codes for the hTERP protein. These functions of telomerase RNA may be involved in maintaining cellular homeostasis as short telomeres result from non-functional telomerase, the defects in mitochondria transport of hTERC and the absence of hTERP protein may play a role in the regulation of signaling pathways and cellular metabolism.

In the current study, we demonstrated that cells deficient in hTERP exhibited enhanced autophagy under conditions of AICAR treatment and autophagy was reduced when amino acids were scarce. Amino acids deprivation leads to mTORC1 inhibition and autophagy stimulation. Meanwhile, AICAR activates AMPK and induces autophagy (Kim et al., 2016) as a result of AICAR treatment leading to decreased binding of class III phosphoinositide 3 (PI3) kinase to beclin-1, which counteracts and reverses the positive effect of AMPK activity on autophagy (Viana et al., 2008). Moreover, AMPK-induced activation of AKT leads to stimulation of compensatory pro-survival mechanisms and the inhibition of autophagy (Leclerc et al., 2010; Zhao et al., 2017). In order to identify the signaling pathway in which hTERP participated, we analyzed the phosphorylation status of proteins from signaling pathways regulated by AMPK and AKT in cells deficient of hTERP under conditions of 2-DG treatment-induced glycolysis inhibition. This compound is a glucose derivative that is phosphorylated by hexokinase 2 and produces 2-DG-phosphate (2-DG-P). The phosphorylated product is then trapped within the cell and cannot be used in subsequent steps of glycolysis, resulting in the accumulation of 2-DG-P and inhibition of hexokinase 2. This results in the inhibition of glycolysis and depletion of ATP. The depletion of ATP leads to the activation of AMPK, stimulation of autophagy, and inhibition of protein synthesis. The 2-DG treatment induces phosphorylation of AKT and its downstream targets in an AMPK-independent manner (Zhong et al., 2008).

We observed decreased levels of phosphorylation of AMPK, but no differences in phosphorylation of AKT. The overexpression of hTERP-3HA in cells deficient in hTERP resulted in the phosphorylation of AMPK being restored to wild-type level. In addition, we observed increased phosphorylation of ULK1 at Ser757 in hTERP-deficient cells, which is known to occur during mTORC1 activation and autophagy inhibition. Increased phosphorylation of ULK1 Ser555 and TSC2 Ser1387, which are downstream targets of AMPK, were also detected in cells deficient in hTERP. AMPK and mTOR kinases are involved in regulating the signaling pathways that control autophagy, protein biosynthesis, cell growth, and cell proliferation (Mihaylova and Shaw, 2011; Porta et al., 2014). Furthermore, energy deficiency is known to stimulate autophagy (Condon and Sabatini, 2019). The inhibition of glycolysis by 2-DG stimulates AMPK activity, which results in the activation of autophagy and inhibition of cellular proliferation (Hardie et al., 2012). Taken together, these data indicate that hTERP is involved in the regulation of AMPK and mTORC1 activity.

The design of experiments performed in this study was made in a way to discriminate between hTERP and hTERC function. We performed the knockout of short RNA fragment that results in the absence of hTERP ORF translation, but not eliminate whole *hTERC* gene. To restore the level of hTERP in knockout cells we expressed it from the fragment with the sequence corresponding to the hTERP ORF, but not the full length hTERC or hTERC-53. We detected similar effects in telomerase positive HEK293T and telomerase negative U2OS cells that use the alternative mechanism of telomere lengthening. All these arguments strongly argue that indeed protein hTERP is involved in the regulation of AMPK- and mTORC1-signaling.

**FIGURE 5:**
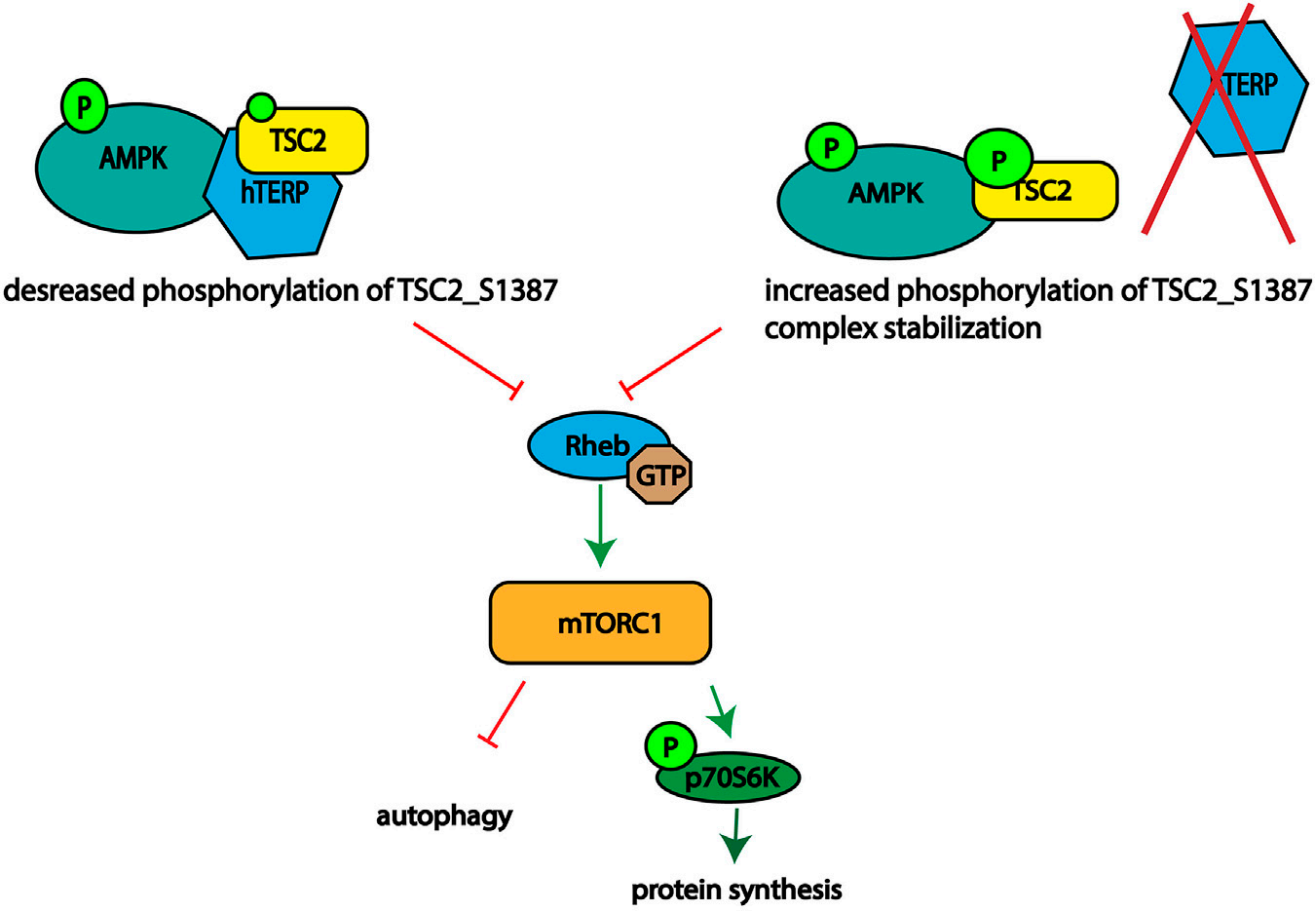
Schematic illustrating the role of hTERP in the regulation of TSC2 phosphorylation at Ser1387 by AMPK.

Based on our data we propose mechanism of action of hTERP in AMPK-mTORC1 signaling (Figure 5). We observed disturbance of the signaling pathway axis. Activation of AMPK should result in phosphorylation of TSC2 at Ser1387 and inhibition of mTORC1 followed by decreased phosphorylation of p70S6K1. We demonstrated activation of mTORC1 and increased phosphorylation of p70S6K1 in cells deficient or overexpressed hTERP. However, the phosphorylation of AMPK was decreased in cells deficient by hTERP and increased when hTERP was overexpressed, but the phosphorylation status of TSC2 at Ser1387 was the opposite of the expected. It was decreased in cells overexpressed hTERP and increased in cells where hTERP was absent. Taken together these findings we suggest that hTERP regulates the phosphorylation of TSC2 at Ser1387 by AMPK and its subsequent interaction and activation of Rheb. We propose that hTERP may be involved in the regulation of interaction between AMPK and TSC2 that leads to the decreased phosphorylation of the last protein. At the same time, the absence of hTERP stimulates the protein’s interaction and phosphorylation of TSC2 leading to the complex stabilization. Stable interaction of TSC2 with AMPK will lead to the inability of TSC2 to stimulate the GTPase activity of Rheb and activation of mTORC1 as a result.

Our findings reveal the connection between telomerase RNA biogenesis and function in telomerase and beyond and signaling pathways that is important for metabolism switching associated with the accelerated proliferation of cells in healthy and pathological processes. The molecular mechanism of hTERP function in the regulation of cellular metabolism is a promising subject for further investigation.

## Data Availability

The original contributions presented in the study are included in the article/Supplementary Material, further inquiries can be directed to the corresponding author.

## References

[B1] Almeida L. Lochner M. Berod L. Sparwasser T. (2016). Metabolic Pathways in T Cell Activation and Lineage Differentiation. Semin. Immunol. 28, 514–524. 10.1016/j.smim.2016.10.009 27825556

[B2] Artandi S. E. Alson S. Tietze M. K. Sharpless N. E. Ye S. Greenberg R. A. (2002). Constitutive Telomerase Expression Promotes Mammary Carcinomas in Aging Mice. Proc. Natl. Acad. Sci. 99, 8191–8196. 10.1073/pnas.112515399 12034875 PMC123043

[B3] Blasco M. A. Funk W. Villeponteau B. Greider C. W. (1995). Functional Characterization and Developmental Regulation of Mouse Telomerase RNA. Science 269, 1267–1270. 10.1126/science.7544492 7544492

[B4] Burgess R. J. Agathocleous M. Morrison S. J. (2014). Metabolic Regulation of Stem Cell Function. J. Intern. Med. 276, 12–24. 10.1111/joim.12247 24697828 PMC4119467

[B5] Cheng H. Fan X. Lawson W. E. Paueksakon P. Harris R. C. (2015). Telomerase Deficiency Delays Renal Recovery in Mice after Ischemia-Reperfusion Injury by Impairing Autophagy. Kidney Int. 88, 85–94. 10.1038/ki.2015.69 25760322 PMC4490111

[B6] Cheng Y. Liu P. Zheng Q. Gao G. Yuan J. Wang P. (2018). Mitochondrial Trafficking and Processing of Telomerase RNA TERC. Cel Rep. 24, 2589–2595. 10.1016/j.celrep.2018.08.003 30184494

[B7] Chow T. T. Zhao Y. Mak S. S. Shay J. W. Wright W. E. (2012). Early and Late Steps in Telomere Overhang Processing in normal Human Cells: the Position of the Final RNA Primer Drives Telomere Shortening. Genes Dev. 26, 1167–1178. 10.1101/gad.187211.112 22661228 PMC3371406

[B8] Condon K. J. Sabatini D. M. (2019). Nutrient Regulation of mTORC1 at a Glance. J. Cel Sci. 132, jcs222570. 10.1242/jcs.222570 PMC6857595 31722960

[B9] Dibble C. C. Cantley L. C. (2015). Regulation of mTORC1 by PI3K Signaling. Trends Cel Biol. 25, 545–555. 10.1016/j.tcb.2015.06.002 PMC4734635 26159692

[B10] Feng J. Funk W. D. Wang S.-S. Weinrich S. L. Avilion A. A. Chiu C.-P. (1995). The RNA Component of Human Telomerase. Science 269, 1236–1241. 10.1126/science.7544491 7544491

[B11] Ferrara-Romeo I. Martinez P. Saraswati S. Whittemore K. Graña-Castro O. Thelma Poluha L. (2020). The mTOR Pathway Is Necessary for Survival of Mice with Short Telomeres. Nat. Commun. 11, 1168. 10.1038/s41467-020-14962-1 32127537 PMC7054554

[B12] Fumagalli M. Rossiello F. Clerici M. Barozzi S. Cittaro D. Kaplunov J. M. (2012). Telomeric DNA Damage Is Irreparable and Causes Persistent DNA-Damage-Response Activation. Nat. Cel Biol. 14, 355–365. 10.1038/ncb2466 PMC3717580 22426077

[B13] González A. Hall M. N. Lin S.-C. Hardie D. G. (2020). AMPK and TOR: The Yin and Yang of Cellular Nutrient Sensing and Growth Control. Cel Metab. 31, 472–492. 10.1016/j.cmet.2020.01.015 32130880

[B14] Hanahan D. Weinberg R. A. (2011). Hallmarks of Cancer: The Next Generation. Cell 144, 646–674. 10.1016/j.cell.2011.02.013 21376230

[B15] Hardie D. G. Ross F. A. Hawley S. A. (2012). AMPK: a Nutrient and Energy Sensor that Maintains Energy Homeostasis. Nat. Rev. Mol. Cel Biol. 13, 251–262. 10.1038/nrm3311 PMC5726489 22436748

[B16] Harris R. C. Cheng H. (2016). Telomerase, Autophagy and Acute Kidney Injury. Nephron 134, 145–148. 10.1159/000446665 27376761 PMC5547439

[B17] Hewitt G. Jurk D. Marques F. D. M. Correia-Melo C. Hardy T. Gackowska A. (2012). Telomeres Are Favoured Targets of a Persistent DNA Damage Response in Ageing and Stress-Induced Senescence. Nat. Commun. 3, 708. 10.1038/ncomms1708 22426229 PMC3292717

[B18] Holczer M. Hajdú B. Lőrincz T. Szarka A. Bánhegyi G. Kapuy O. (2019). A Double Negative Feedback Loop between mTORC1 and AMPK Kinases Guarantees Precise Autophagy Induction upon Cellular Stress. Int. J. Mol. Sci. 20, 5543. 10.3390/ijms20225543 PMC6888297 31703252

[B19] Holz M. K. Ballif B. A. Gygi S. P. Blenis J. (2005). mTOR and S6K1 Mediate Assembly of the Translation Preinitiation Complex through Dynamic Protein Interchange and Ordered Phosphorylation Events. Cell 123, 569–580. 10.1016/j.cell.2005.10.024 16286006

[B20] Hoxhaj G. Manning B. D. (2020). The PI3K-AKT Network at the Interface of Oncogenic Signalling and Cancer Metabolism. Nat. Rev. Cancer 20, 74–88. 10.1038/s41568-019-0216-7 31686003 PMC7314312

[B21] Huang J. Dibble C. C. Matsuzaki M. Manning B. D. (2008). The TSC1-TSC2 Complex Is Required for Proper Activation of mTOR Complex 2. Mol. Cel. Biol. 28, 4104–4115. 10.1128/MCB.00289-08 PMC2423120 18411301

[B22] Inoki K. Li Y. Xu T. Guan K.-L. (2003). Rheb GTPase Is a Direct Target of TSC2 GAP Activity and Regulates mTOR Signaling. Genes Dev. 17, 1829–1834. 10.1101/gad.1110003 12869586 PMC196227

[B23] Inoki K. Zhu T. Guan K.-L. (2003). TSC2 Mediates Cellular Energy Response to Control Cell Growth and Survival. Cell 115, 577–590. 10.1016/S0092-8674(03)00929-2 14651849

[B24] Kabeya Y. (2000). LC3, a Mammalian Homologue of Yeast Apg8p, Is Localized in Autophagosome Membranes after Processing. EMBO J. 19, 5720–5728. 10.1093/emboj/19.21.5720 11060023 PMC305793

[B25] Kim J. Yang G. Kim Y. Kim J. Ha J. (2016). AMPK Activators: Mechanisms of Action and Physiological Activities. Exp. Mol. Med. 48, e224. 10.1038/emm.2016.16 27034026 PMC4855276

[B26] Kowarz E. Löscher D. Marschalek R. (2015). Optimized Sleeping Beauty Transposons Rapidly Generate Stable Transgenic Cell Lines. Biotechnol. J. 10, 647–653. 10.1002/biot.201400821 25650551

[B27] Kwiatkowski D. J. Manning B. D. (2005). Tuberous Sclerosis: a GAP at the Crossroads of Multiple Signaling Pathways. Hum. Mol. Genet. 14, R251–R258. 10.1093/hmg/ddi260 16244323

[B28] Leclerc G. M. Leclerc G. J. Fu G. Barredo J. C. (2010). AMPK-induced Activation of Akt by AICAR Is Mediated by IGF-1R Dependent and Independent Mechanisms in Acute Lymphoblastic Leukemia. J. Mol. Signal. 5, 15. 10.1186/1750-2187-5-15 20863384 PMC2955666

[B29] Lingner J. Cooper J. P. Cech T. R. (1995). Telomerase and DNA End Replication: No Longer a Lagging Strand Problem? Science 269, 1533–1534. 10.1126/science.7545310 7545310

[B30] Löffler A. S. Alers S. Dieterle A. M. Keppeler H. Franz-Wachtel M. Kundu M. (2011). Ulk1-mediated Phosphorylation of AMPK Constitutes a Negative Regulatory Feedback Loop. Autophagy 7, 696–706. 10.4161/auto.7.7.15451 21460634

[B31] Menon S. Dibble C. C. Talbott G. Hoxhaj G. Valvezan A. J. Takahashi H. (2014). Spatial Control of the TSC Complex Integrates Insulin and Nutrient Regulation of mTORC1 at the Lysosome. Cell 156, 771–785. 10.1016/j.cell.2013.11.049 24529379 PMC4030681

[B32] Mihaylova M. M. Shaw R. J. (2011). The AMPK Signalling Pathway Coordinates Cell Growth, Autophagy and Metabolism. Nat. Cel Biol. 13, 1016–1023. 10.1038/ncb2329 PMC3249400 21892142

[B33] Monaghan P. Ozanne S. E. (2018). Somatic Growth and Telomere Dynamics in Vertebrates: Relationships, Mechanisms and Consequences. Phil. Trans. R. Soc. B 373, 20160446. 10.1098/rstb.2016.0446 29335370 PMC5784066

[B34] Olovnikov A. M. (1973). A Theory of Marginotomy. J. Theor. Biol. 41, 181–190. 10.1016/0022-5193(73)90198-7 4754905

[B35] Pfeiffer V. Lingner J. (2012). TERRA Promotes Telomere Shortening through Exonuclease 1-mediated Resection of Chromosome Ends. Plos Genet. 8, e1002747. 10.1371/journal.pgen.1002747 22719262 PMC3375253

[B36] Porta C. Paglino C. Mosca A. (2014). Targeting PI3K/Akt/mTOR Signaling in Cancer. Front. Oncol. 4, 64. 10.3389/fonc.2014.00064 24782981 PMC3995050

[B37] Ran F. A. Hsu P. D. Wright J. Agarwala V. Scott D. A. Zhang F. (2013). Genome Engineering Using the CRISPR-Cas9 System. Nat. Protoc. 8, 2281–2308. 10.1038/nprot.2013.143 24157548 PMC3969860

[B38] Roake C. M. Artandi S. E. (2020). Regulation of Human Telomerase in Homeostasis and Disease. Nat. Rev. Mol. Cel Biol. 21, 384–397. 10.1038/s41580-020-0234-z PMC7377944 32242127

[B39] Roake C. M. Chen L. Chakravarthy A. L. Ferrell J. E. Raffa G. D. Artandi S. E. (2019). Disruption of Telomerase RNA Maturation Kinetics Precipitates Disease. Mol. Cel 74, 688–700.e3. 10.1016/j.molcel.2019.02.033 PMC6525023 30930056

[B40] Roh J.-i. Kim Y. Oh J. Kim Y. Lee J. Lee J. (2018). Hexokinase 2 Is a Molecular Bridge Linking Telomerase and Autophagy. PLOS ONE 13, e0193182. 10.1371/journal.pone.0193182 29462198 PMC5819818

[B41] Rubtsova M. Dontsova O. (2020). Human Telomerase RNA: Telomerase Component or More? Biomolecules 10, 873. 10.3390/biom10060873 PMC7355840 32517215

[B42] Rubtsova M. Naraykina Y. Vasilkova D. Meerson M. Zvereva M. Prassolov V. (2018). Protein Encoded in Human Telomerase RNA Is Involved in Cell Protective Pathways. Nucleic Acids Res. 46, 8966–8977. 10.1093/nar/gky705 30102362 PMC6158713

[B43] Rubtsova M. P. Vasilkova D. P. Moshareva M. A. Malyavko A. N. Meerson M. B. Zatsepin T. S. (2019). Integrator Is a Key Component of Human Telomerase RNA Biogenesis. Sci. Rep. 9, 1701. 10.1038/s41598-018-38297-6 30737432 PMC6368637

[B44] Salminen A. Kaarniranta K. (2012). AMP-activated Protein Kinase (AMPK) Controls the Aging Process via an Integrated Signaling Network. Ageing Res. Rev. 11, 230–241. 10.1016/j.arr.2011.12.005 22186033

[B45] Ségal-Bendirdjian E. Geli V. (2019). Non-canonical Roles of Telomerase: Unraveling the Imbroglio. Front. Cel Dev. Biol. 7, 332. 10.3389/fcell.2019.00332 PMC6914764 31911897

[B46] Shackelford D. B. Shaw R. J. (2009). The LKB1-AMPK Pathway: Metabolism and Growth Control in Tumour Suppression. Nat. Rev. Cancer 9, 563–575. 10.1038/nrc2676 19629071 PMC2756045

[B47] Skeen J. E. Bhaskar P. T. Chen C.-C. Chen W. S. Peng X.-d. Nogueira V. (2006). Akt Deficiency Impairs normal Cell Proliferation and Suppresses Oncogenesis in a P53-independent and mTORC1-dependent Manner. Cancer Cell 10, 269–280. 10.1016/j.ccr.2006.08.022 17045205

[B48] Tseng C.-K. Wang H.-F. Burns A. M. Schroeder M. R. Gaspari M. Baumann P. (2015). Human Telomerase RNA Processing and Quality Control. Cel Rep. 13, 2232–2243. 10.1016/j.celrep.2015.10.075 26628367

[B49] Viana R. Aguado C. Esteban I. Moreno D. Viollet B. Knecht E. (2008). Role of AMP-Activated Protein Kinase in Autophagy and Proteasome Function. Biochem. Biophysical Res. Commun. 369, 964–968. 10.1016/j.bbrc.2008.02.126 18328803

[B50] Watanabe R. Wei L. Huang J. (2011). mTOR Signaling, Function, Novel Inhibitors, and Therapeutic Targets. J. Nucl. Med. 52, 497–500. 10.2967/jnumed.111.089623 21421716

[B51] Zhao Y. Hu X. Liu Y. Dong S. Wen Z. He W. (2017). ROS Signaling under Metabolic Stress: Cross-Talk between AMPK and AKT Pathway. Mol. Cancer 16, 79. 10.1186/s12943-017-0648-1 28407774 PMC5390360

[B52] Zhong D. Liu X. Schafer-Hales K. Marcus A. I. Khuri F. R. Sun S.-Y. (2008). 2-Deoxyglucose Induces Akt Phosphorylation via a Mechanism Independent of LKB1/AMP-Activated Protein Kinase Signaling Activation or Glycolysis Inhibition. Mol. Cancer Ther. 7, 809–817. 10.1158/1535-7163.MCT-07-0559 18413794

