# Supplementary material for: Human Telomerase RNA Protein Encoded by Telomerase RNA is Involved in Metabolic Responses
Source: Front Cell Dev Biol. 2021 Dec 7;9:754611. doi: 10.3389/fcell.2021.754611 (PMC8688989; doi:10.3389/fcell.2021.754611)
Supplement: Supplementary file 1 [file Table1.DOCX]

**Supplementary Figure 1.** Characterization of hTERP expression in wild type and modified HEK293T cells. (**A**) Verification of knockout at genomic level. Upper panel (wt) corresponds to the sequence of PCR fragment obtained from genomic DNA of wild type HEK293T cells. Lower panel (dhTERP) corresponds to the sequence of PCR fragment obtained from genomic DNA of knock-outed HEK293T cells. (**B**) Samples prepared from HEK293T overexpressing hTERP cells, wild type HEK293T cells, knockouted HEK293T cells overexpressing hTERP and knockouted HEK293T cells for hTERP were probed with anti-hTERP antibodies. GAPDH was used as a loading control. **(C)** Samples prepared from HEK293T cells exogenously expressing 3HA (HA) and hTERP-3HA (hTERP) were probed with anti-HA antibodies. −d, expression was not induced by doxycycline treatment; +d, cells were treated with doxycycline to induce the integrated cassette. GAPDH was used as a loading control. **(D)** Samples prepared from U2OS cells exogenously expressing 3HA (HA) and hTERP-3HA (hTERP) were probed with anti-HA antibodies. −d, expression was not induced by doxycycline treatment, +d, cells were treated with doxycycline to induce the integrated cassette. GAPDH was used as a loading control.
